# ElectroCom61: A multiclass dataset for detection of electronic components

**DOI:** 10.1016/j.dib.2025.111331

**Published:** 2025-01-27

**Authors:** Md. Faiyaz Abdullah Sayeedi, Anas Mohammad Ishfaqul Muktadir Osmani, Taimur Rahman, Jannatul Ferdous Deepti, Raiyan Rahman, Salekul Islam

**Affiliations:** a Department of Computer Science and Engineering, United International University, Bangladesh; b Department of Electrical and Computer Engineering, North South University, Bangladesh

**Keywords:** Electronic component, Deep learning, Image processing, Object detection, Computer vision, E-waste management, Industrial manufacturing, Industrial automation

## Abstract

In contemporary industrial, robotics, and technical education settings, the efficient detection and sorting of electronic components play a pivotal role in advancing automation and increasing efficiency in these sectors. To address this need, we present “ElectroCom61,” a comprehensive multi-class object detection dataset encompassing 61 commonly used electronic components. Our dataset, sourced from the electronic components collection at United International University (UIU) in Dhaka, Bangladesh, comprises 2121 meticulously annotated images. We ensured that these images reflect real-world conditions, incorporating varied lighting, backgrounds, distances, and camera angles to bolster the potential machine learning model's robustness. We also divided the dataset into training, validation, and test sets to facilitate deep learning model development. Additionally, we conducted minimal pre-processing to optimise model training and performance. “ElectroCom61” stands as a valuable asset for developing cutting-edge electronic component detection systems, with far-reaching applications in both education and industry. Its potential applications span from interactive educational tools to e-waste management systems and streamlined inventory management processes in electronic manufacturing and automation. The code for technical validation of this dataset is available on GitHub: https://github.com/faiyazabdullah/ElectroCom61

Specifications Table

**Table:** 

Subject	Computer Vision and Pattern Recognition
Specific subject area	Detection of electronic components ranging from simple single-purpose circuit components to more complex ones.
Type of data	Image Raw, Processed
Data collection	The data are collected by capturing images using the regular camera of three different smartphone devices.
Data source location	Institution: United International University (UIU) City/Region: Dhaka Country: Bangladesh The images were taken in the Electronics Lab Support Room (Room 524) at United International University.
Data accessibility	Repository name: Mendeley Data Data identification number: 10.17632/6scy6h8sjz.2 Direct URL to data: https://data.mendeley.com/datasets/6scy6h8sjz/2

## 1 Value of the Data

• The dataset offers images captured under varied lighting conditions, against multiple backgrounds, and multiple camera angles. This diversity prepares deep learning models for real-world applications where electronic components might be encountered in low lighting and on various backgrounds, enhancing the models' accuracy and robustness in diverse operational settings.
• With 61 distinct classes of electronic components ranging from basic elements like resistors and capacitors to complex devices such as Arduino boards and GSM modules, the dataset serves as a comprehensive resource for training and validating deep learning models. This extensive coverage facilitates the creation of systems that can recognise and differentiate a wide array of electronic parts which can be crucial for automated sorting and assembly operations.
• The dataset is organised into training, validation, and test sets, which supports effective machine learning practices. This structure allows models to be thoroughly trained, fine-tuned, and evaluated, helping to ensure they learn from a wide variety of data and can perform well on new, unseen examples.
• The dataset is ideal for developing and refining technologies used in automated inventory and quality control systems within electronics manufacturing, sorting, and retail. By training models capable of recognising a vast array of components, these systems can become more efficient, reducing labour costs and minimising human error.
• By providing a dataset that includes a wide variety of components under different environmental conditions, ElectroCom61 encourages further research in computer vision and deep learning fields. Researchers can explore new algorithms and techniques for image recognition, object detection, object segmentation, and image classification, advancing the state-of-the-art in automated electronic component handling and identification.
• The dataset holds significant promise for advancing e-waste management systems. By training deep learning models on this dataset, researchers can develop tools capable of automatically identifying electronic components, facilitating efficient e-waste recycling processes.

## 2 Background

The “ElectroCom61” dataset was developed in response to a growing need for improved automated systems in the electronics industry [1,2], both to help in identifying and sorting various electronic components [[3], [4], [5], [6]], as well as the recycling and proper disposal of discarded components [[7], [8], [9], [10]]. This dataset is meant to assist in computer vision technologies, where accurate classification of objects through image analysis stands as a crucial capability. It also aims to advance research in automated component recognition [11], which is essential for enhancing production efficiency and quality control; and to contribute to the development of more sophisticated e-waste management systems [12]. This initiative will facilitate computer vision technologies, where the accurate classification of objects through image analysis stands as a crucial capability [[13], [14], [15]].

In creating “ElectroCom61” [16], we acknowledge the gap in existing datasets by analysing available datasets as well as the ones used in works done in computer vision based real time detection [12,17,18] and classification [17,[19], [20], [21]]. The concerns arose that datasets are often not publicly available or only available upon request [2,18,22], existing ones do not have a diversity in objects [3,11], are specialised on a type of component [2,11,23] or include copyrighted materials [24]. We address all three of these concerns by incorporating images under different lighting conditions and against diverse backgrounds. Our dataset is designed to challenge and refine the robustness of current object detection models. This compilation not only supports theoretical advancements in deep learning methodologies [[25], [26], [27]] but also offers practical benefits for technological applications. A brief comparison of related datasets is covered in Table 1 to highlight the gap we mean to address.

**Table 1:** Gap Analysis.

Dataset	Diversity	No. of Classes	Availability	No. of Images	No. of Annotations	Comments
[2]	Specialised	4	Private	1000	43,160	200 Images were augmented to 1000
[3]	Specialised	9	Public	500	N/A	Specialized in batteries
[11]	Specialised	3	Public	328	N/A	
[18]	Specialised	4	Upon Request	11,875	28,536	
[22]	Diverse	20	Private	12,000	N/A	Detection dataset but no. of annotations was not mentioned
[23]	Specialised	2024	Public	748	9313	Specialized in ICs in PCBs
[24]	Diverse	36	Public	11,000	N/A	Includes copyrighted images
Ours	Diverse	61	Public	2121	12,937	

In educational settings, this dataset could be used to create interactive learning tools, enhancing hands-on training in electronic components identification. In manufacturing, it will support automated sorting systems to streamline inventory and quality control [5,17]. For e-waste management, the dataset will facilitate efficient classification and separation of components, promoting sustainable recycling practices [9,17].

## 3 Data Description

### 3.1 Data collection

**Fig. 1:**
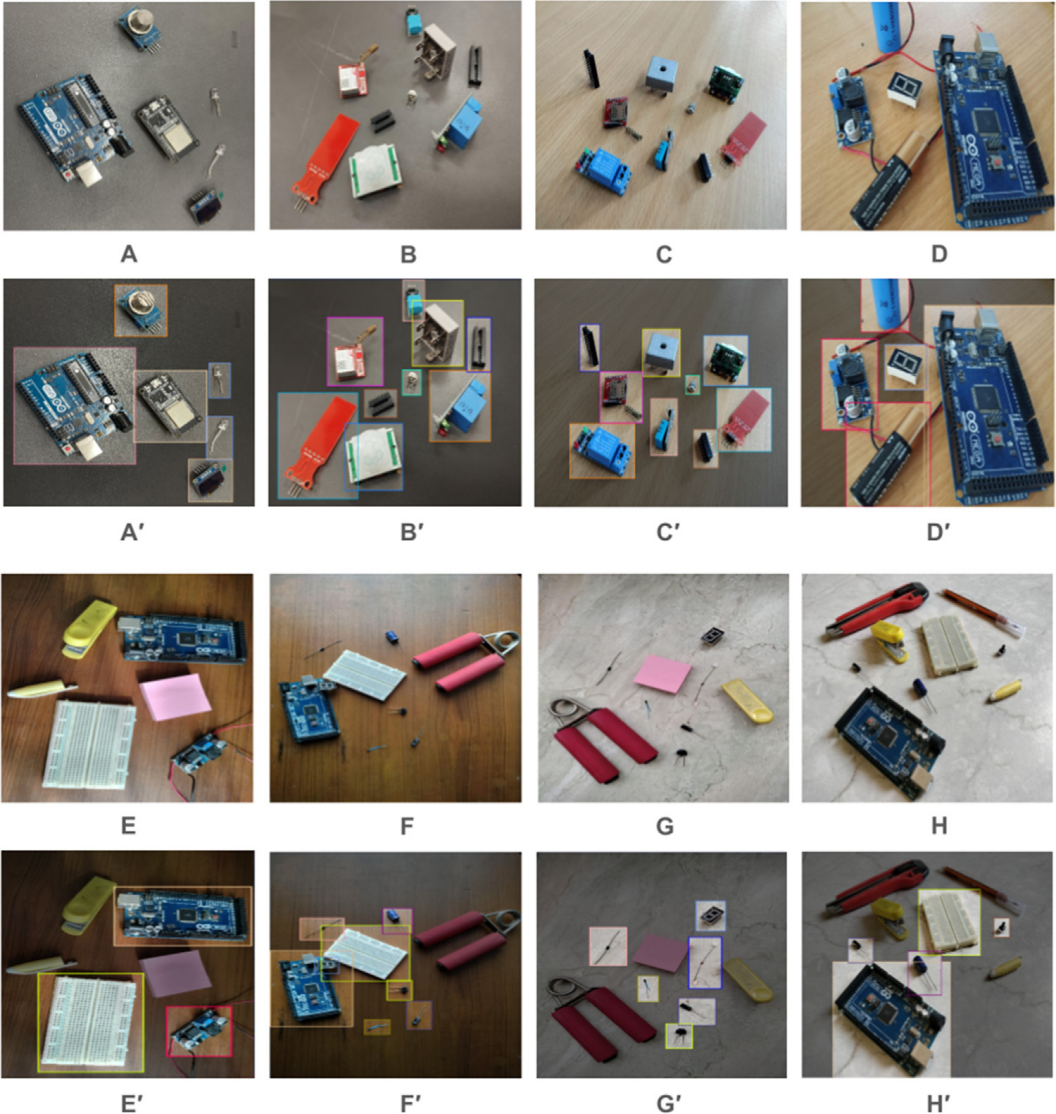
Example of Fully Annotated Images using Bounding Boxes. (A, B, C, D) Sample Images of controlled environment. (E, F, G, H) Sample Images of more complex, less controlled environment, where electronic components are surrounded by unrelated objects or materials.

In the initial phase of our project, data collection for the ElectroCom61 dataset involved meticulous fieldwork. The dataset was collected in the controlled environment of the Electronics Lab Support Room (Room 524) at the United International University (UIU), located in Dhaka, Bangladesh. This comprehensive data collection was achieved through the use of main cameras on three smartphones. The use of multiple devices ensures a diverse representation of imaging qualities and perspectives, capturing a realistic range of conditions that electronic components might be subjected to in practical scenarios. Table 2 shows the camera specifications for all the cameras used to capture the images. The dataset is reliably annotated using the tool Roboflow [28], which guarantees that the images are authentic and representative. This careful process ensures that the dataset is effective for real-time electronic component detection. Fig. 1 shows an overview of the annotated dataset.

**Table 2:** Camera Specifications Table.

Device	Capacity	Display	Camera
Smartphone 1	128 GB	Type: AMOLED Size: 6.43 inches, 99.8 cm2 (∼85.3 % screen-to-body ratio) Resolution: 1080 x 2400 pixels, 20:9 ratio (∼409 ppi density) Protection: Schott Xensation Up Always-on display	Triple: 64 MP, f/1.8, (wide), PDAF 2 MP, f/2.4, (macro) 2 MP, f/2.4, (depth) Features: LED flash, HDR, panorama
Smartphone 2	128 GB	Type: AMOLED, 120Hz, 1200 nits (peak) Size: 6.67 inches, 107.4 cm2 (∼85.0 % screen-to-body ratio) Resolution: 1080 x 2400 pixels, 20:9 ratio (∼395 ppi density) Protection: Corning Gorilla Glass 3	Triple: 48 MP, f/1.8, (wide), PDAF 8 MP, f/2.2, 120˚ (ultrawide), 1/4.0", 1.12µm 2 MP, f/2.4, (macro) Features: Dual-LED dual-tone flash, HDR, panorama
Smartphone 3	256 GB	Type: Super Retina XDR OLED, HDR10, Dolby Vision, 625 nits (HBM), 1200 nits (peak) Size: 5.4 inches, 71.9 cm2 (∼85.1 % screen-to-body ratio) Resolution: 1080 x 2340 pixels, 19.5:9 ratio (∼476 ppi density) Protection: Ceramic Shield glass	Dual: 12 MP, f/1.6, 26mm (wide), 1.4µm, dual pixel PDAF, OIS 12 MP, f/2.4, 13mm, 120˚ (ultrawide), 1/3.6" Features: Dual-LED dual-tone flash, HDR (photo/panorama)

### 3.2 Data preprocessing

Before usage in machine learning workflows, the images underwent several preprocessing steps within Roboflow to ensure the quality and effectiveness of a dataset for training machine learning models. The primary steps include - (a) Auto-Orient: Images were automatically oriented based on the embedded sensor data to maintain consistency in the dataset. (b) Resize: All images were resized to a uniform dimension of 640 × 640 pixels. This stretching maintains aspect ratio consistency and ensures that all input data into machine learning models are of the same dimension, which is crucial for the consistency of feature extraction processes in deep learning algorithms.

### 3.3 Distribution analysis

**Fig. 2:**
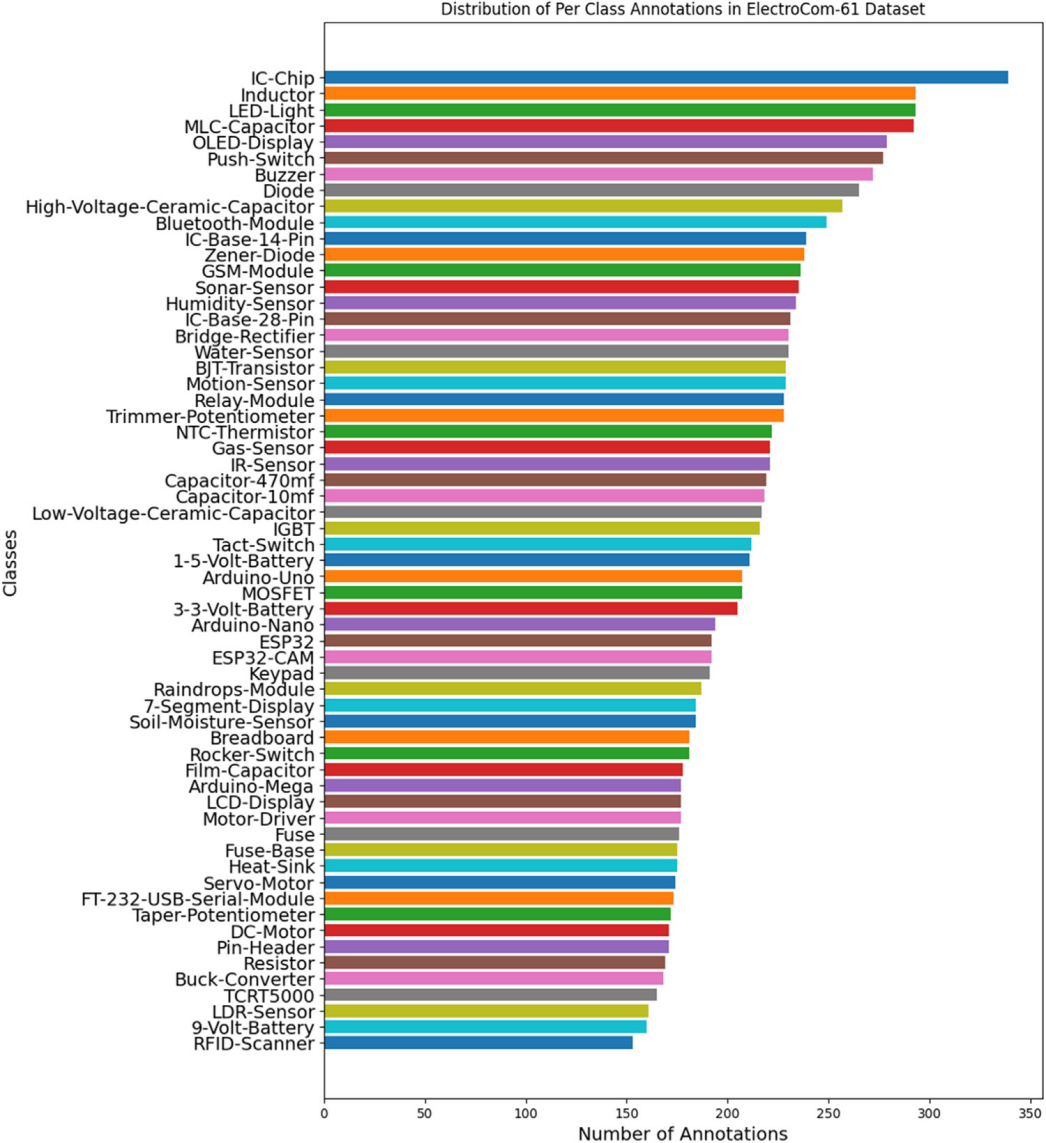
Distribution of Per Class Annotation in ElectroCom61 dataset.

The “ElectroCom61” dataset includes a total of 2121 images and 12,937 annotations, which are distributed across various classes representing different types of electronic components. The distribution is split into 70 % (1478 images) training, 20 % (438 images) validation, and 10 % (205 images) testing, ensuring a comprehensive range for model training while leaving sufficient data for model validation and testing. This structured split aids in mitigating the model's overfitting and underfitting by providing diverse examples during training and unbiased evaluation during testing. The list of classes and the per-class annotation in the dataset is shown in Fig. 2. The classes are also quite balanced having a normal distribution among the classes where the class having the highest annotation (IC-chip) has only twice the number of annotations compared to the class with the lowest annotations (RFID scanner).

### 3.4 Folder structure

**Fig. 3:**
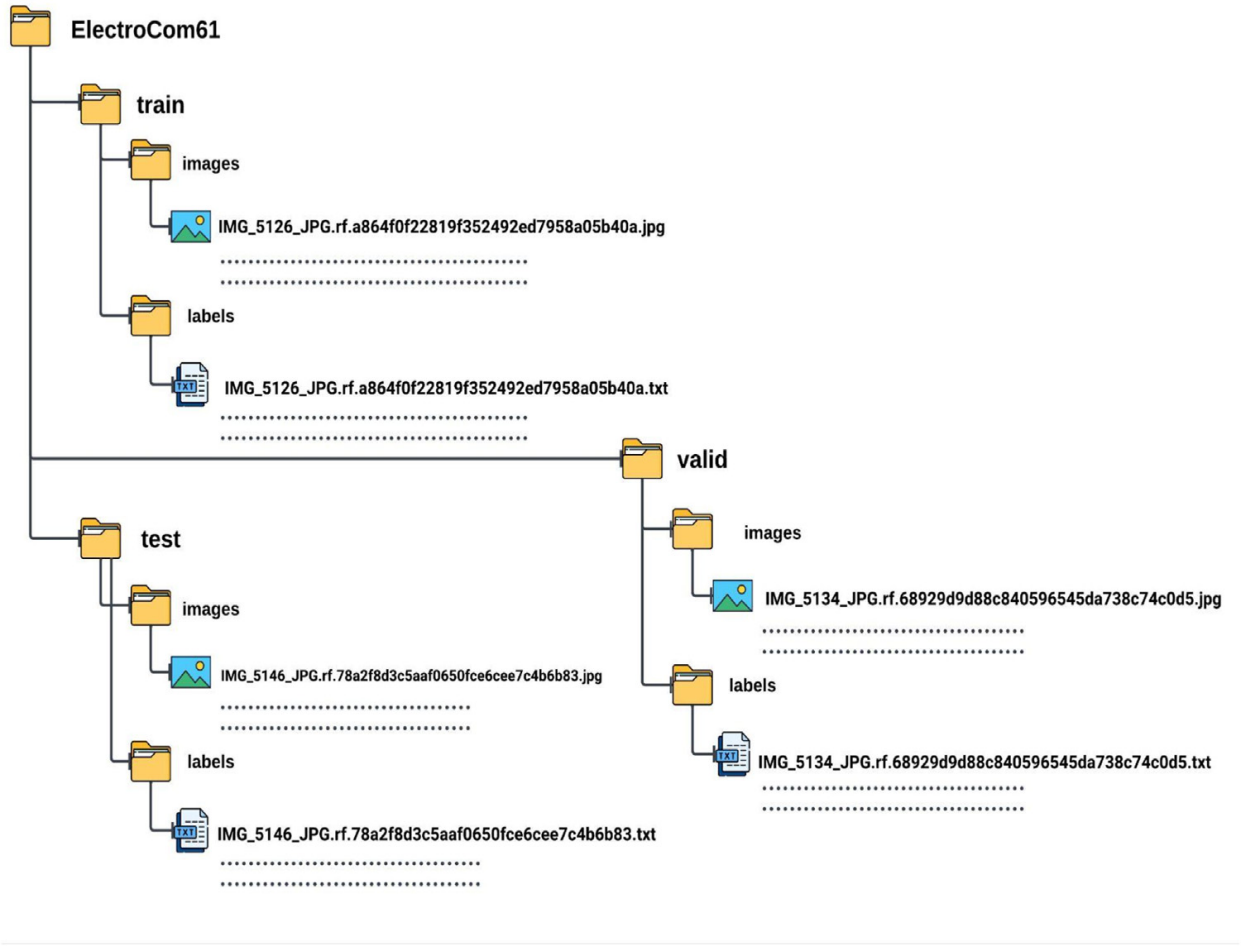
Folder Structure of ElectroCom61 Dataset.

The dataset is organised into three primary directories corresponding to the data splits: Train, Valid, and Test. Each directory contains two subdirectories - (a) Image: This folder contains the actual images of the electronic components. Each image file is named uniquely to prevent any naming conflicts and ensure easy traceability. (b) Label: This folder contains the annotations corresponding to each image in the 'image' folder. These annotations are stored in text files and include details such as the class labels and bounding box coordinates for object detection tasks. The folder structure is shown in Fig. 3.

### 3.5 Metadata

In addition to the main dataset, we provide a CSV file named “Metadata_ElectroCom61.csv” which offers detailed metadata for each image in the dataset. The file includes several fields: IMAGE_NAME, which identifies the file; TYPE_OF_OBJECTS, a numerical value denoting the number of unique electronic components present in the image; OBJECT_NAME, specifying the exact name of the component(s) present in the image, where the preceding number followed by an underscore (_) denotes the number of occurrences of that specific component; BACKGROUND, describing the specific type of background present against the electronic components; DEVICE_NAME, the smartphone used to capture the image; and DATA_TYPE, indicating whether the image belongs to the Train, Valid, or Test set. This metadata file serves as a valuable resource for researchers who wish to analyse the dataset more deeply, enabling them to filter and sort the data based on specific attributes and conditions.

## 4 Experimental Design, Materials and Methods

In this section, we discuss the experimental design and technical validation of the dataset.

### 4.1 Experimental design

Our experimental design focused on creating a diverse and comprehensive dataset of electronic components that were reflective of real-world variability in a laboratory setting. A total of 61 different electronic components, ranging from commonly used items like capacitors and diodes to specialized sensors, were selected from the hardware department at the university. These components were photographed between 12:00 PM and 2:00 PM to leverage natural sunlight for better image clarity and quality.

Photographs were taken in three distinct environments to introduce background and lighting diversity, enhancing the robustness of the dataset: (a) Wooden Desk (b) Ceramic Floor (c) Dark Desk. This variety in backgrounds was intended to simulate the practical challenges faced in image processing applications, such as identifying components against complex or reflective surfaces. For instance, small and dark components are less visible on a dark desk, and reflective surfaces can sometimes be miscategorised as the glass in a fuse.

The photographic approach included taking images from multiple angles—including bird's eye views and close-ups—particularly for smaller components that required detailed visibility. Some images featured isolated components to avoid overlap, while others depicted realistic scenarios with overlapping components to mimic real-life settings. Additionally, we incorporated real-world scenarios where electronic components are surrounded by unrelated objects or materials, further enhancing the dataset's diversity and realism. This approach not only helped in capturing the components in various configurations but also added to the complexity of the dataset, making it more representative of actual conditions.

Components were photographed in groups of 6 to 12 at a time. The selection of these groups was managed through a randomization algorithm implemented in Python, ensuring a balanced representation of each class. This method helped maintain an unbiased data collection process and addressed any underrepresentation by revisiting the collection process for classes with fewer images.

After data collection, the dataset underwent a cleaning process to remove any invalid data entries. Subsequently, the cleaned images were annotated with precision to ensure accurate labels reflecting each component's details. These annotations were critical in preparing the dataset for further processing. Then the data was pre-processed using various techniques to enhance the quality and usability of machine learning applications. The prepared data was then split into training, validation, and testing sets managed by Roboflow, ensuring that the dataset was ready for deployment in machine learning models without manual bias in data handling.

**Fig. 4:**
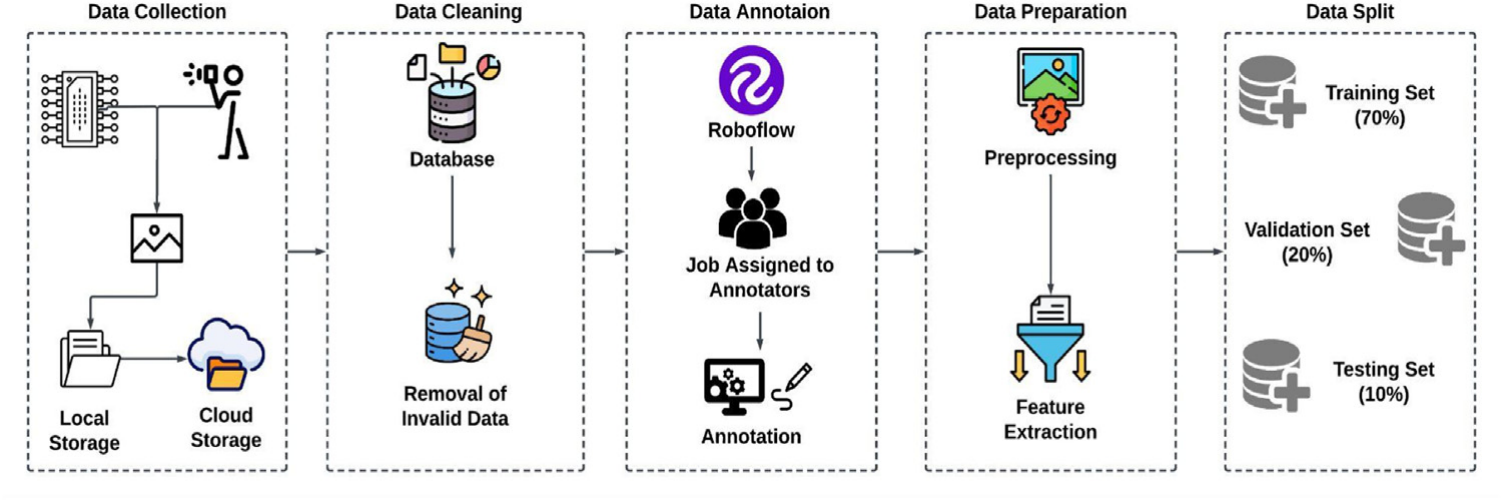
Framework of Experimental Design/Methodology.

This structured approach not only ensured the diversity and quality of our dataset but also prepared it for rigorous applications in machine learning, specifically in automated electronic component detection and sorting systems. The overall experimental design is shown in Fig. 4.

### 4.2 Technical validation

For the technical validation of our dataset, we utilized two state-of-the-art deep learning models: YOLOv9s [29] and EfficientDetV2 [30], both known for their efficiency and accuracy in object detection tasks. YOLOv9s, previously trained on the MS COCO dataset, provided a robust pre-trained foundation for feature recognition, while EfficientDetV2 is widely acclaimed for its precision and efficiency in similar applications. We trained both models using our dataset, focusing on the training set images, and subsequently evaluated their performance using the validation and testing sets.

The effectiveness of the model was quantified through several key performance metrics. For YOLOv9s, the mean Average Precision (mAP) at an Intersection over Union (IoU) threshold of 50 % was measured at 95.9 %, with a precision of 92.4 %, recall of 94.9 %, total loss of 0.5795, and F1-score of 93.63 %. EfficientDetV2 achieved a mAP@50 of 95.6 %, with a higher precision of 95.6 %, recall of 99.7 %, and F1-score of 97.6 % and less total loss of 0.40146. These results indicate both models’ high accuracy and reliability in detecting and classifying electronic components from the images, with EfficientDetV2 showing slightly higher performance in precision and recall. This strong performance highlights the dataset's quality and applicability in real-world scenarios. Table 3 shows the summary of technical validation.

**Table 3:** Technical Validation Table.

Model	mAP@50	Precision	Recall	F1-Score	Total Loss
YOLOv9s	95.9 %	92.4 %	94.9 %	93.63 %	0.5795
EfficientDetV2	95.6 %	95.6 %	99.7 %	97.6 %	0.40146

**Fig. 5:**
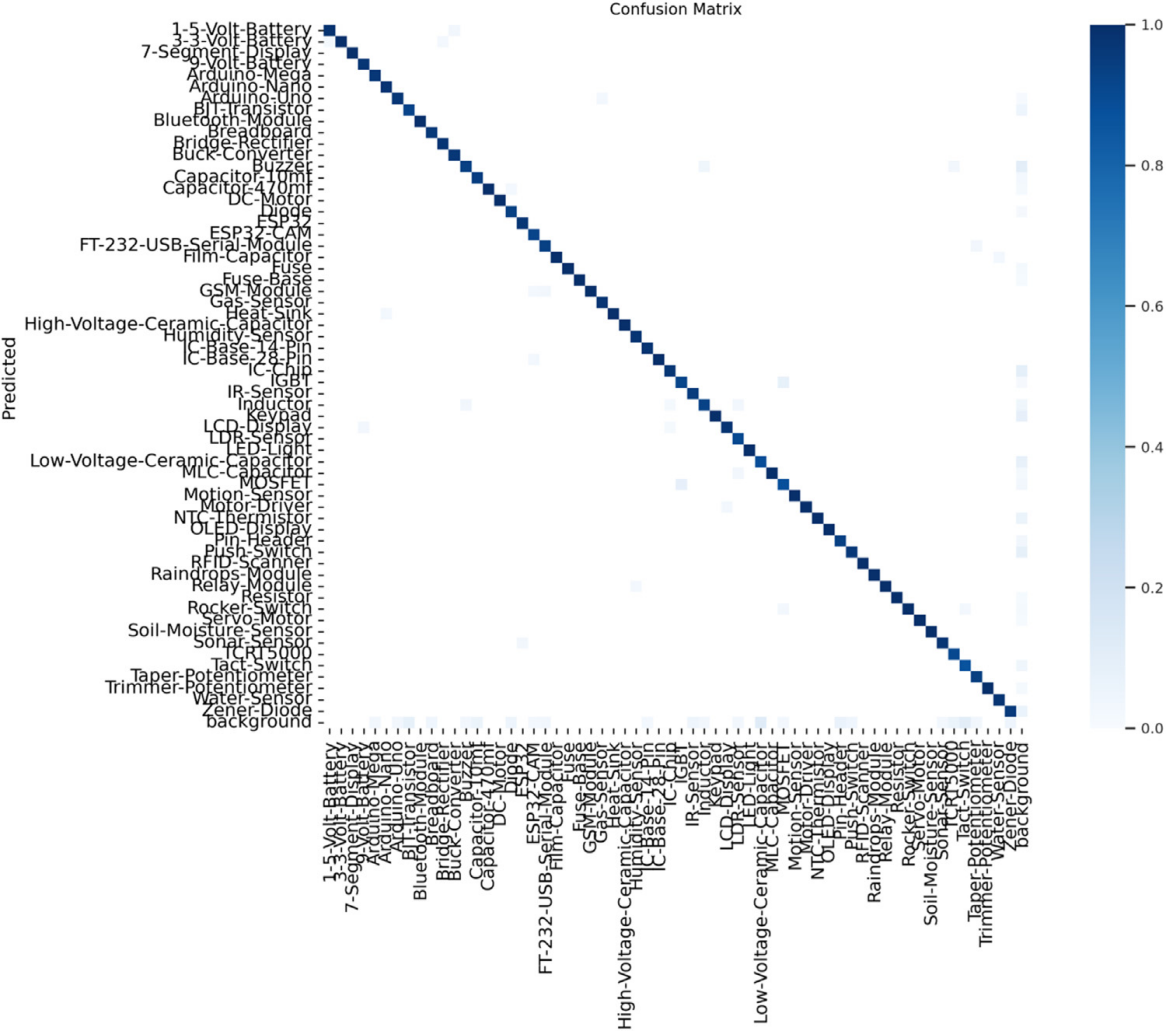
Confusion Matrix for YOLOv9s.

**Fig. 6:**
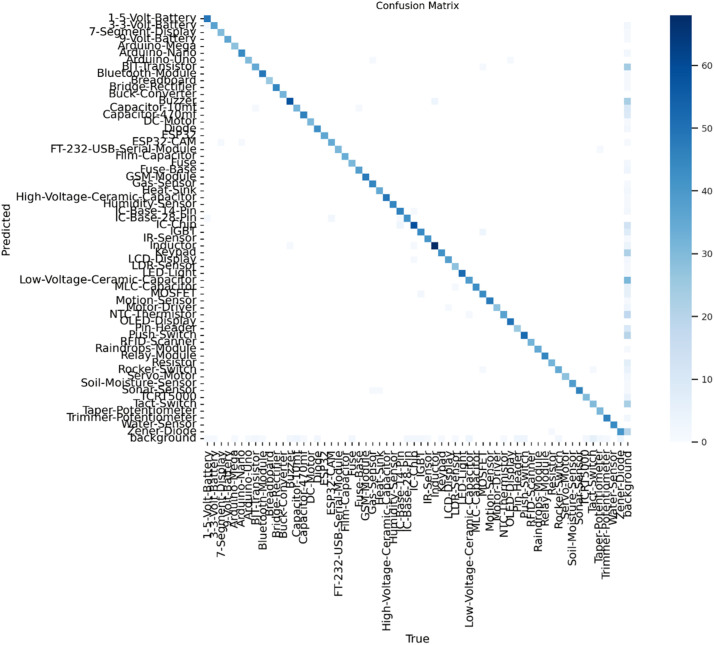
Confusion Matrix for EfficientDetV2.

The confusion matrices for both the YOLOv9s (Fig. 5) and EfficientDetV2 (Fig. 6) models on this dataset demonstrate strong performance, with predictions aligning closely along the diagonal, reflecting high accuracy in classifying the 61 electronic components. Both models showed minor misclassifications, likely due to visual similarities among certain components, such as capacitors and resistors, which affected precision slightly. The well-defined background class in both models helped reduce false positives, contributing to reliable detection. However, EfficientDetV2’s higher recall suggests a stronger capacity for identifying almost all relevant objects, making it particularly robust in terms of detection sensitivity. Overall, both models demonstrated effective and balanced performance, with YOLOv9s excelling in balanced metrics and EfficientDetV2 in recall, positioning them as suitable choices for real-time electronic component recognition tasks.

## Limitations

The potential distortion due to resizing images to maintain a uniform size of 640 × 640 pixels may alter the original aspect ratio of the components, leading to a less accurate recognition of diverse shapes and sizes. When electronic components with specific shapes and aspect ratios are reshaped disproportionately, details that are crucial distinguishing similar items may be lost altered, reducing the model's ability to recognize fine-grained features. Additionally, resizing can lead to the loss of contextual information (e.g., relative size or component detail), potentially lowering precision in component classification and increasing misclassification rates, especially for components with similar visual characteristics. Even though we included unrelated objects and materials, some limitations remain. Specifically, when larger unrelated objects appear alongside smaller target components, the likelihood of misclassification increases. This highlights a challenge in achieving full environmental realism in the dataset.

## Ethics Statement

This work does not involve human subjects, animal experiments, or data derived from social media platforms. Permission for data collection was obtained from the authority of the Electronic Lab Support Room at United International University, ensuring compliance with institutional standards. In alignment with ethical data collection protocols, all images were captured in controlled environments, strictly avoiding any inclusion of human faces or identifying features to ensure privacy. We followed strict guidelines to prevent any sensitive objects like – personal belongings, confidential documents, computer/mobile screens from appearing in the dataset. Additionally, we ensured that proprietary information was visible in the images, and data collection was conducted during designated hours to minimize disruptions and maintain a professional setting. The dataset was created with transparency and integrity, adhering to ethical standards and best practices for data collection and handling.

## CRediT authorship contribution statement

**Md. Faiyaz Abdullah Sayeedi:** Conceptualization, Methodology, Software, Data curation, Formal analysis, Visualization, Writing – original draft. **Anas Mohammad Ishfaqul Muktadir Osmani:** Conceptualization, Methodology, Software, Data curation, Formal analysis, Visualization, Writing – original draft. **Taimur Rahman:** Conceptualization, Methodology, Software, Data curation, Formal analysis, Visualization, Writing – original draft. **Jannatul Ferdous Deepti:** Software, Data curation. **Raiyan Rahman:** Conceptualization, Methodology, Formal analysis, Visualization, Writing – review & editing, Supervision. **Salekul Islam:** Formal analysis, Validation, Writing – review & editing, Supervision.

## Data Availability

Mendeley DataElectroCom61: A Multiclass Dataset for Detection of Electronic Components (Original data).
